# Effects of Wind Exposure and Deficit Irrigation on Vegetative Growth, Yield Components and Berry Composition of Malbec and Cabernet Sauvignon

**DOI:** 10.3390/plants13101292

**Published:** 2024-05-08

**Authors:** Rodrigo Alonso, Flavio Muñoz, Rubén Bottini, Patricia Piccoli, Federico J. Berli

**Affiliations:** 1 Grupo de Bioquímica Vegetal, Instituto de Biología Agrícola de Mendoza (IBAM), Consejo Nacional de Investigaciones Científicas y Técnicas—Facultad de Ciencias Agrarias, Universidad Nacional de Cuyo, Almirante Brown 500, Chacras de Coria, Mendoza 5507, Argentina; flavio_m500@hotmail.com (F.M.); ppiccoli@fca.uncu.edu.ar (P.P.); fberli@fca.uncu.edu.ar (F.J.B.); 2 Instituto Argentino de Veterinaria, Ambiente y Salud (IAVAS), Universidad Juan Agustín Maza, Av. Acceso Este Lateral Sur 2245, Guaymallén, Mendoza 5519, Argentina; rbottini48@gmail.com

**Keywords:** environmental factors, management strategy, mechanically-induced stress, phenotypic plasticity, terroir, *Vitis vinifera* L.

## Abstract

The impact of global warming on Argentine viticulture may result in a geographical shift, with wine-growing regions potentially moving towards the southwest, known as one of the windiest regions in the world. Deficit irrigation is a widely used strategy to control the shoot growth and improve fruit quality attributes, such as berry skin polyphenols. The present study aimed to assess the effects of different wind intensities and irrigation levels, as well as their interactions, on field-grown *Vitis vinifera* L. cvs. Malbec and Cabernet Sauvignon. The experiment was conducted during two growing seasons with two wind treatments (sheltered and exposed) and two irrigation treatments (well-watered and moderate deficit irrigation) in a multifactorial design. Vegetative growth, stomatal conductance, shoot biomass partition, fruit yield components and berry skin phenolics were evaluated. Our study found that, generally, wind exposure reduced vegetative growth, and deficit irrigation increased the proportion of smaller berries within the bunches. Meanwhile, deficit irrigation and wind exposure additively increased the concentration of berry skin phenolics. Combined stressful conditions enhance biomass partition across the shoot to fruits in Malbec, increasing the weight of bunches and the number of berries. Our findings offer practical implications for vineyard managers in windy regions, providing actionable insights to optimize grapevine cultivation and enhance wine quality.

## 1. Introduction

Argentina ranks second in the Americas and seventh globally in terms of vineyard surface [1]. Argentinian viticulture extends from 22° to 45° South latitude, predominantly along the piedmont of the Andes Mountains, characterized by a semi-arid continental climate. Precipitation levels are inadequate to sustain the vine cycle, which necessitates irrigation. Climate projections for the next 50–75 years due to global warming suggest a potential shift of wine-growing regions towards the southwest and higher altitudes [2]. Patagonia, the southernmost wine-growing region worldwide, accounts for less than 3% of Argentina’s total vineyard area [3]. Generally, the region features a continental, temperate climate with significant thermal amplitude [4]. Moreover, Patagonia is renowned as one of the windiest regions in the world, particularly during spring and summer. In North Patagonia (above 40° S), these seasons experienced the highest frequency and wind events exceeding 5 m/s, with gusts reaching up to 33 m/s [5].

Plants exposed to wind undergo physiological responses that affect the leaf boundary layer, consequently influencing crucial gas exchange processes [6]. Additionally, wind acts as an environmental signal that moves the aerial parts of the plants, producing mechanically induced stress (MIS) [7]. One of the most noticeable effects of MIS is a reduction in shoot length, often accompanied by increased radial growth. This phenomenon, termed “thigmomorphogenesis” by Jaffe [8], has been observed in various species, including grapevines [9,10]. Also, wind can cause physical damage to plants through abrasion, leaf stripping, and sandblasting [11].

In general, research on the effects of wind has predominantly focused on forest trees and herbaceous species. However, less attention has been given to fruit crops despite the presumably economic consequences [10]. While our understanding of the impact of wind on grapevines remains limited, it is worth noting that constant winds with moderate (3 to 6 m/s) to strong (>6 m/s) intensities are integral components of the terroir in viticulture regions such as the Salinas Valley in California, the Swan Valley in Australia, and the Western Cape Province in South Africa, among others [12,13,14].

In the limited studies conducted, vines exposed to wind have shown lower stomatal conductance, reduced shoot length and total leaf area [9,12,13,15,16]. Additionally, some researchers have observed differential responses to wind among different grapevine cultivars and interactions with other environmental conditions. Kobriger et al. [16] found a common response to moderate and strong winds of five cultivars, a decreased transpiration rate, and differences in their recovery after the wind ceased. Campbell-Clause [12] observed that wind increased stomatal resistance for two grapevine cultivars and an interaction between the cultivar and the sunlight levels.

Regarding yield components, dissimilar results are found in the current literature, possibly due to the use of different varieties and/or experimental systems. Freeman et al. [13] and Dry et al. [9] reported that wind can reduce grapevine yield, contrary to Pienaar [14]. Furthermore, divergent findings have been reported regarding the influence of wind on the content of berry phenolic compounds [9,14], which are secondary metabolites known to confer protection against various environmental stressors [17]. Phenolic compounds play a crucial role in red wine quality characteristics, such as color, astringency and bitterness [18].

Deficit Irrigation (DI) represents a common irrigation management strategy in arid climates, involving the application of less water than what a vineyard loses via evapotranspiration during a portion of the growing season. The primary objective of DI is to regulate water supply to control shoot growth and to enhance fruit quality attributes, including the concentration of berry skin phenolics compounds [19,20]. However, our previous research has shown that applying moderate DI in high-altitude vineyards, i.e., exposed to elevated solar ultraviolet-B (UV-B) radiation, had adverse effects on yield and certain wine quality characteristics [21,22]. In essence, the impact of DI can vary depending on the climatic conditions of a particular wine region and/or season. To our knowledge, there are no scientific reports on the effects of DI in a vineyard influenced by moderate and strong winds.

Phenotypic plasticity, characterized by an organism’s ability to modify its phenotype in response to varying environments, represents a quantitative trait for mitigating environmental negative impacts [23]. A high degree of phenotypic plasticity was found for *Vitis vinifera* cv. Malbec [24], which is the iconic cultivar in Argentina with the largest cultivated area [3]. Conversely, Cabernet Sauvignon, a globally recognized grapevine cultivar and the fourth most planted in Argentina [3] has demonstrated greater consistency across diverse environments [25].

Based on the intricate interplay between cultivars and environmental factors, recognized collectively as terroir, it is relevant to explore the impact of wind on cultivars exhibiting differential phenotypic plasticity. Furthermore, it is important for winegrowers to evaluate the effects of implementing DI as a management strategy in vineyards exposed to moderate to strong winds. The aim of this study was to assess the impact of varying wind intensities and irrigation levels, as well as their interactions, on quali-quantitative traits of field-grown *Vitis vinifera* L. in a comparative analysis between Malbec and Cabernet Sauvignon.

## 2. Results

### 2.1. Vegetative Growth, Stomatal Conductance, Biomass Partitioning, Fruit Yield and Proportion of Berry Sizes

**Figure 1:**
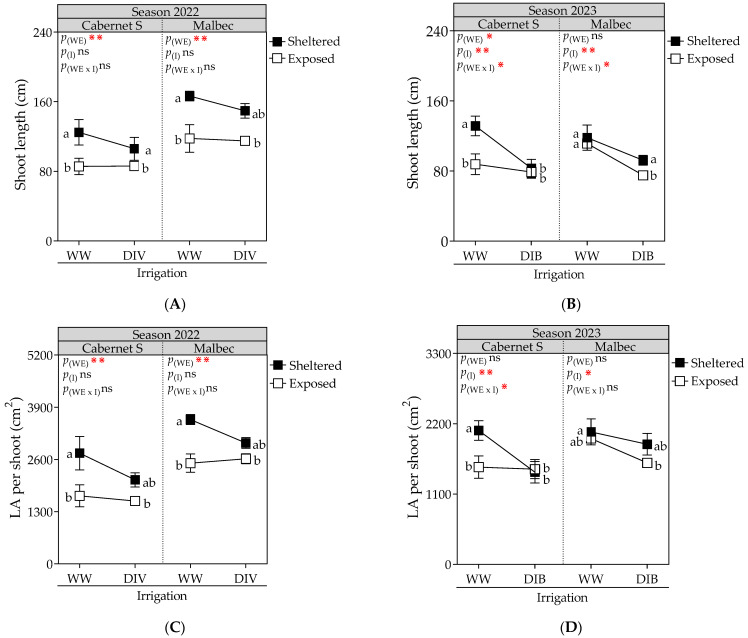
Shoot length and leaf area (LA) per shoot, measured at harvest, in Cabernet Sauvignon and Malbec vines exposed to combined wind exposure (WE) and irrigation (I) treatments during season 2021–2022 (**A**,**C**) and season 2022–2023 (**B**,**D**). Values are means for each factor combination ±SEM (n = 6), and different letters indicate significant differences (Fisher’s LSD, *p* ≤ 0.05). ⋇, ⋇⋇, and ns indicate significance at *p* < 0.1, 0.05, and not significant, respectively. p_(WE)_ and p_(I)_: effects of WE and I, respectively; p_(WE×I)_: interaction effect of factors. WW, well-watered; DIV, moderate water deficit from veraison to harvest; DIB, moderate water deficit from budburst to harvest.

The vegetative growth variables were significantly influenced by wind exposure, DI, and their interactions (Figure 1). During the first season of the study, wind-exposed vines of CS and Mb exhibited reductions in shoot length (23.5% and 25.3%, respectively) and leaf area per shoot (30.5% and 19.5%, respectively), while the DI treatment from veraison to harvest did not affect plant growth (Figure 1A,C). Conversely, during the second season, wind exposure and DI interacted, resulting in differences between cultivars. Notably, for CS, sheltered vines under WW treatment showed a marked increase in vegetative growth (43.4%, on average) compared to the exposed/WW treatment. In the case of Mb, the promotion of shoot length and leaf area was observed for the WW treatment, irrespective of the wind, compared to the DI treatment (Figure 1B,D).

**Figure 2:**
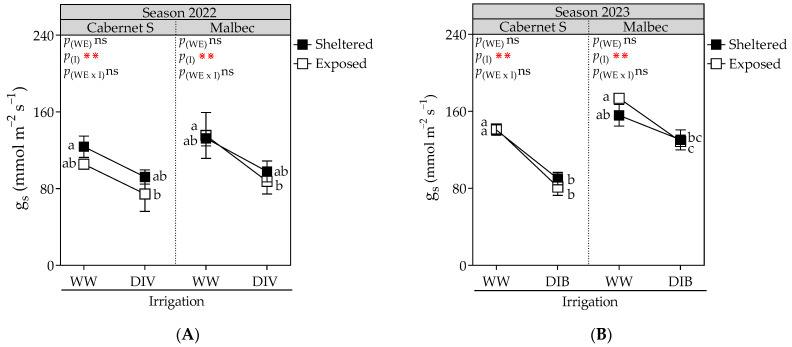
Stomatal conductance (gs) measured at harvest in Cabernet Sauvignon and Malbec vines exposed to combined wind exposure (WE) and irrigation (I) treatments during season 2021–2022 (**A**) and season 2022–2023 (**B**). Values are means for each factor combination ±SEM (n = 6), and different letters indicate significant differences (Fisher’s LSD, *p* ≤ 0.05). ⋇⋇, and ns indicate significance at *p* < 0.05, and not significant, respectively. p_(WE)_ and p_(I)_: effects of WE and I, respectively; p_(WE×I)_: interaction effect of factors. WW, well-watered; DIV, moderate water deficit from veraison to harvest; DIB, moderate water deficit from budburst to harvest.

Stomatal conductance exhibited a significant decrease in vines subjected to DI treatments during both seasons (DIV and DIB; Figure 2). On average, Mb and CS grapevines under DIV and DIB exhibited a reduction of 30.2% compared to WW treatment.

**Figure 3:**
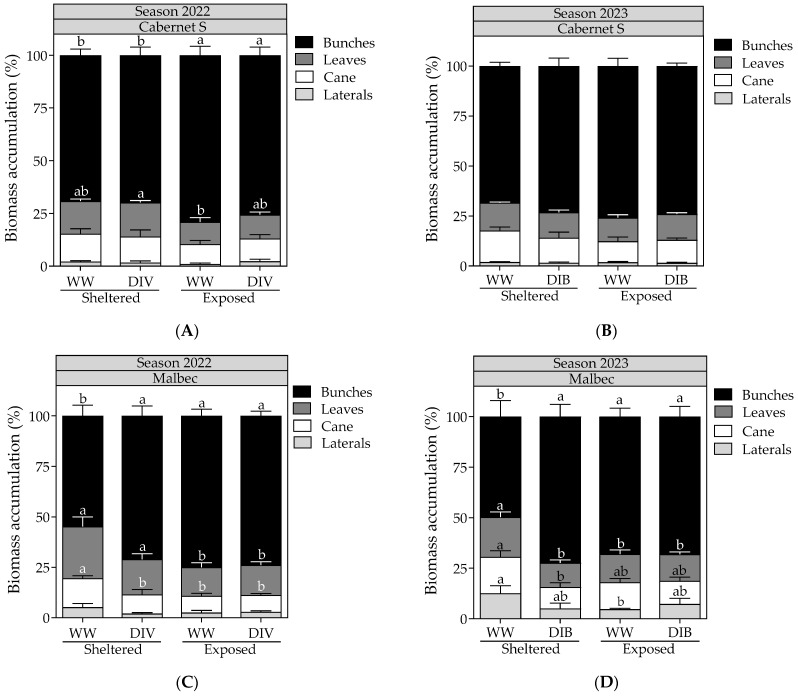
Total shoot biomass accumulation, measured at harvest in Cabernet Sauvignon and Malbec vines exposed to combined wind exposure (WE) and irrigation (I) treatments during season 2021–2022 (**A**,**C**) and season 2022–2023 (**B**,**D**). Values are means for each factor combination ±SEM (n = 6), and different letters within each organ indicate statistically significant differences between treatments (Fisher’s LSD, *p* ≤ 0.05). WW, well-watered; DIV, moderate water deficit from veraison to harvest; DIB, moderate water deficit from budburst to harvest.

As shown in Figure 3A, CS vines exposed to wind represented, on average, 77.4% of the total biomass accumulation in bunches. Conversely, CS vines subjected to wind-sheltered treatments constituted 69.5% of the total, reflecting a 7.9% reduction. No significant treatment effects were observed in CS grapevines during the 2023 season (Figure 3B). Illustrated in Figure 3C, WW and wind-sheltered Mb grapevines showed that 54.8% of the total biomass accumulation was attributed to bunches, representing the treatment with the most significant reduction in bunch biomass (18.5%). Similar results were observed in Mb grapevines during the 2023 season (Figure 3D).

**Figure 4:**
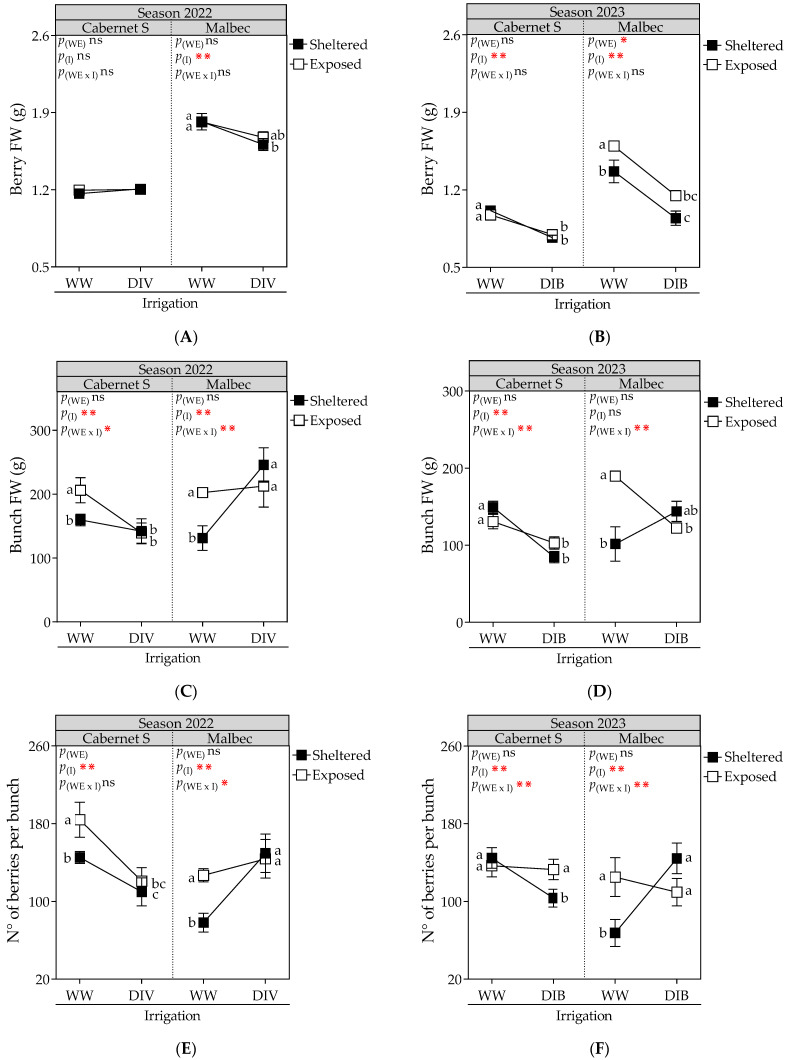
Berry fresh weight (FW), bunch FW and number of berries per bunch, measured at harvest, in Cabernet Sauvignon and Malbec vines exposed to combined wind exposure (WE) and irrigation (I) treatments during season 2021–2022 (**A**,**C**,**E**) and season 2022–2023 (**B**,**D**,**F**). Values are means for each factor combination ±SEM (n = 6), and different letters indicate significant differences (Fisher’s LSD, *p* ≤ 0.05). ⋇, ⋇⋇, and ns indicate significance at *p* < 0.1, 0.05, and not significant, respectively. p_(WE)_ and p_(I)_: effects of WE and I, respectively; p_(WE×I)_: interaction effect of factors. WW, well-watered; DIV, moderate water deficit from veraison to harvest; DIB, moderate water deficit from budburst to harvest.

Berry FW was markedly different for cultivars and was affected by DI, except for CS in 2022 (Figure 4A,B). Mb vines were more sensitive to DI (DIV and DIB), leading to a significant decrease in berry FW during both seasons (23.6%, on average). Additionally, wind-exposed Mb plants showed an increased berry FW (17%).

Bunch FW (Figure 4C,D) and the number of berries per bunch (Figure 4E,F) were similarly affected by the treatments. For CS plants, DI resulted in an average decrease of 27.4% in bunch FW and 22.8% in the number of berries per bunch across both seasons. Furthermore, during the 2022 season, wind-exposed grapevines under WW exhibited a significant 29.6% increase in bunch FW and a 26.9% increase in the number of berries, indicating an interaction. For Mb plants, the interaction between wind exposure and irrigation was significant in both seasons, with the combination of wind-sheltered/WW resulting in a 40% reduction in bunch FW and a 45% reduction in the number of berries per bunch.

**Figure 5:**
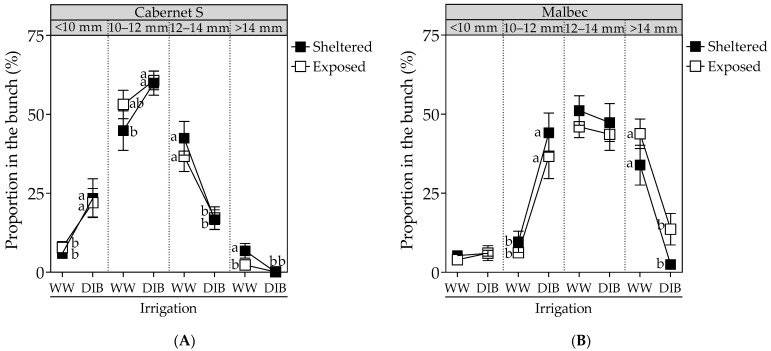
Percent size (%) distribution of berries in the bunch, measured at harvest, in Cabernet Sauvignon (**A**) and Malbec (**B**) vines exposed to combined wind exposure (WE) and irrigation (I) treatments during season 2022–2023. Values are means for each factor combination ±SEM (n = 6), and different letters within each berry size category indicate statistically significant differences between treatments (Fisher’s LSD, *p* ≤ 0.05). WW, well-watered; DIB, moderate water deficit from budburst to harvest.

Regarding the proportion of berry sizes in the bunch, DI similarly affected both cultivars, leading to an increase in smaller berries. Specifically, for CS plants (Figure 5A), DIB increased the proportion of berries with sizes <10 mm and sizes between 10–12 mm by 15.8% and 11.8%, respectively, while markedly reducing the proportion of berries with sizes between 12–14 mm and >14 mm (by 22.4% and 4.4%, respectively). Similarly, for Mb vines (Figure 5B), DIB amplified the proportion of berries with sizes between 10–12 mm by 32.5% while concurrently reducing the proportion of berries >14 mm by 30.8%.

### 2.2. Berry Skin Total Phenolics and Relationship between Total Anthocyanins and Total Soluble Solids

**Figure 6:**
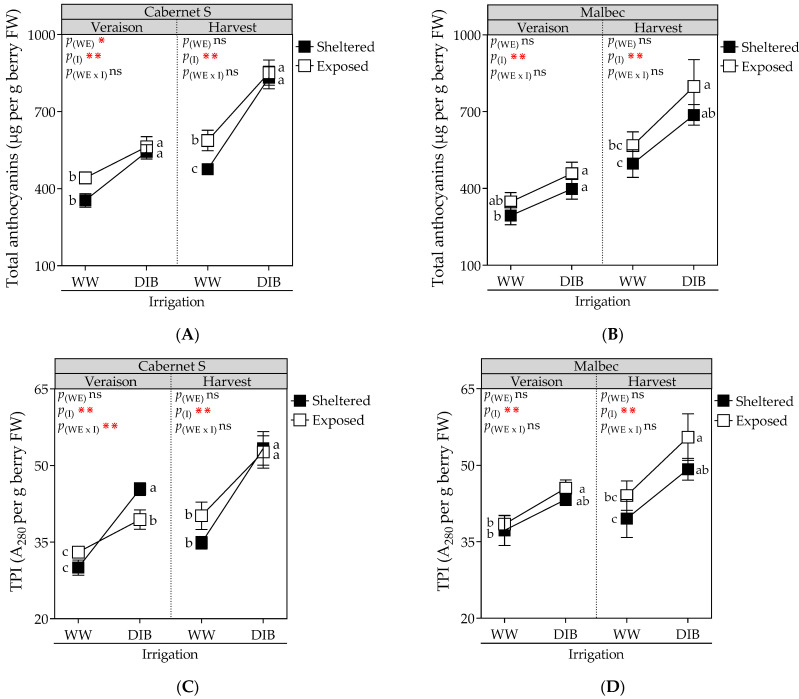
Total anthocyanins (μg per g berry FW) and total polyphenols index (TPI; A_280_ per g berry FW) measured at harvest in Cabernet Sauvignon (**A**,**C**) and Malbec (**B**,**D**) vines exposed to combined wind exposure (WE) and irrigation (I) treatments during season 2022–2023. Values are means for each factor combination ±SEM (n = 6), and different letters within each phenological stage indicate statistically significant differences between treatments (Fisher’s LSD, *p* ≤ 0.05). ⋇, ⋇⋇, and ns indicate significance at *p* < 0.1, 0.05, and not significant, respectively. p_(WE)_ and p_(I)_: effects of WE and I, respectively; p_(WE×I)_: interaction effect of factors. WW, well-watered; DIB, moderate water deficit from budburst to harvest.

Figure 6A,C reveal the impact of DI on the concentration of skin total phenolic of CS grapevines at veraison and harvest during the 2022–2023 growing season. At the harvest stage, the combined effects of wind exposure and DI led to an additive increase in total anthocyanins in CS (79.2% higher compared to sheltered and WW). Additionally, DIB resulted in a 40.5% increase in TPI compared to WW. At harvest, Mb vines exhibited an additive effect between wind exposure and DI, resulting in a 60.6% increase in total anthocyanin and a 41% increase in TPI compared to grapevines that were sheltered and WW (Figure 6B,D).

**Figure 7:**
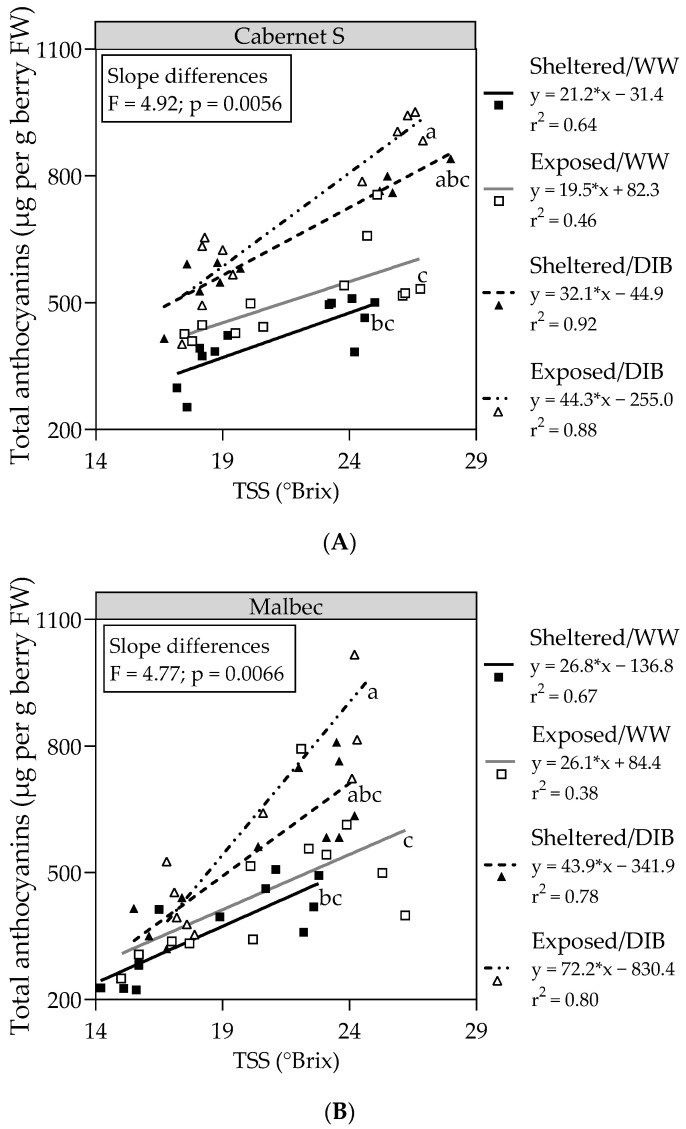
Linear regressions between the total anthocyanins (μg per g berry FW) and total soluble solids (TSS), measured at veraison and harvest in Cabernet Sauvignon (**A**) and Malbec (**B**) vines exposed to combined wind exposure and irrigation treatments during season 2022–2023. Different letters represent significant differences (Fisher’s LSD, *p* ≤ 0.05) between the slopes of each linear regression model. WW, well-watered; DIB, moderate water deficit from budburst to harvest.

Figure 7 presents linear regression models depicting the relationship between TSS and total anthocyanins for Mb and CS, considering veraison and harvest data from the 2023 season. In both cultivars, the steepest slope was observed with the combined treatment Exposed/DIB, significantly differing from the combined treatments Exposed/WW and Sheltered/WW.

## 3. Discussion

Vegetative growth was significantly influenced by wind exposure in both cultivars and both seasons. The significant reduction in vegetative growth of wind-exposed vines aligns with findings from experiments conducted by Dry et al. [9] and Bettiga et al. [15] with field-grown Cabernet Franc and Chardonnay, respectively. Deficit irrigation from veraison (season 2022) did not affect vegetative growth in any of the cultivars. However, during the second season, when deficit irrigation started earlier (from budburst), interaction effects were observed, revealing distinct impacts between the cultivars. CS grapevines WW and wind-sheltered had higher vegetative development, whereas, in Mb grapevines, the combination of both stress conditions (i.e., wind exposure and DI) led to reduced vegetative expression. Of particular significance in grapevines is the phenomenon observed during veraison, where shoot growth undergoes a notable slowdown or cessation. This shift in growth patterns occurs as the plant redirects its resources toward the accumulation of sugars and other compounds within the bunches, as highlighted by Köse et al. [26]. Pre-veraison water stress led to a more pronounced reduction in both shoot and berry growth compared to stress occurring after veraison [27]. This finding suggests a physiological response to water stress that may be contingent upon the developmental stage of the vines.

In the present study, stomatal conductance was only affected by deficit irrigation. Meanwhile, other authors have suggested that wind can induce stomatal closure. Freeman et al. [13] conducted their research in the Salinas Valley, where grapevines’ reduced stomatal conductance was linked to dense morning fog followed by afternoon winds. Kobriger et al. [16] conducted experiments in a controlled environment (phytotron) with vines in pots, and Campbell-Clause [12] demonstrated that in field-grown grapevines, the increases in stomatal resistance were not observed until wind speeds exceeded 5 m/s. Possibly, the lack of wind-induced effects on stomatal conductance in our study was related to the wind speeds during the measurement days (<5 m/s). Considering the established link between decreased stomatal conductance, photosynthesis, and subsequent vegetative growth [28], it is reasonable to speculate that overall wind exposure may have influenced gas exchange.

Matthews and Anderson [29,30] suggested that mild water stress diminishes grapevine vigor, decreases competition for carbohydrates by growing shoot tips, and promotes a shift in the allocation of photoassimilates towards reproductive tissues and secondary metabolites. Given that wind can generate stress conditions similar to mild water stress, it is plausible that it could induce similar effects on grapevine physiology. However, further studies are necessary to investigate this hypothesis. In the current study, regarding the impact of treatments on photoassimilate distribution across the shoot, a differential response was observed between cultivars. In the case of Mb grapevines subjected to lesser stress conditions (WW and wind-sheltered), the most significant reduction in bunch weight (DW and FW) and the number of berries per bunch was observed. Additionally, Pienaar [14] noted in field-grown cv. Merlot that wind-sheltered grapevines produced smaller bunches with fewer berries compared to wind-exposed grapevines. With respect to fruit production, a differential effect of DI was observed between the cultivars. For CS plants, DI resulted in a decreased bunch FW across both seasons, consistent with observations made by Keller et al. [31] and Cassasa et al. [32] in CS grapevines under DI.

Deficit irrigation typically results in smaller berries as it hampers both cell division and cell expansion [33]. Given that anthocyanins and other phenolic compounds accumulate in the berry skin [34], berry size significantly influences wine grape quality. Smaller berries have a relatively greater solute-to-solvent ratio compared to larger berries. In the current study, during the 2023 growing season, where DI was applied from budburst to harvest, both cultivars were similarly affected, irrespective of wind exposure. This led to a higher proportion of smaller berries within the bunches and potentially greater oenological quality.

Our findings reveal a significant impact of combined stress treatment, involving DI from budburst to harvest coupled with wind exposure, on the concentration of berry skin total phenolics. This resulted in an additive increase in total anthocyanin and TPI compared to grapevines that were wind-sheltered and WW. It is widely acknowledged that DI enhances total phenolic compounds due to both concentration effects from reduced berry size and increased synthesis [35], but the effect of wind on grapevine berry skin total phenolic compounds remains relatively understudied. Pienaar [14] observed an augmentation in phenolic content in wind-exposed Merlot grapes; however, Dry et al. [9] did not report any significant effect. Notably, our observations indicate that both Mb and CS vines exposed to wind exhibited an additional increase in key secondary metabolite compounds crucial from an oenological perspective. Considering that enzymes involved in regulating phenolic biosynthesis respond positively to solar radiation [36], possibly wind-exposed vines, which exhibited lower vegetative expression compared to wind-sheltered grapevines, received increased solar radiation on their bunches. This could have contributed to the augmented phenolic expression observed in the exposed vines.

In previous work, with vines under high levels of solar UV-B radiation, we observed that the more stressful conditions treatments present the more prominent slopes in the linear regression between berry sugar and berry skin total anthocyanins, suggesting that sugar reductions may be a response attributable to the cost of forming secondary metabolites to provide protection to plants [37]. In the present study, we observed similar responses in grapevines under the combined treatment wind-exposed/DI, indicating that the observed changes in berry composition may potentially enhance wine quality.

## 4. Materials and Methods

### 4.1. Plant Material and Experimental Design

**Figure A1:**
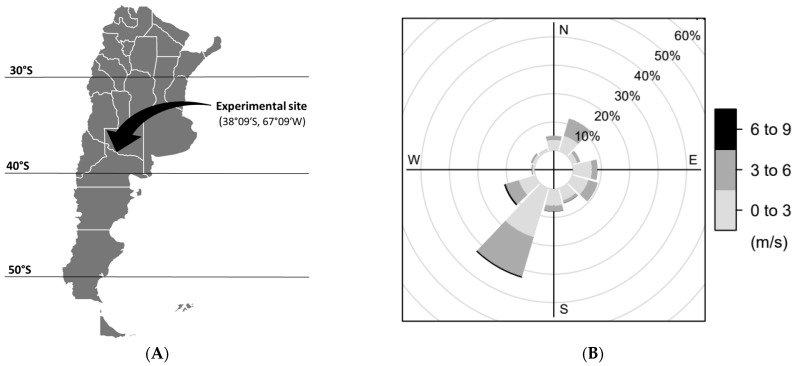
Location of the experimental site on the map of Argentina (**A**). Average wind speed and frequency of counts by wind directions (%) during September to March (grapevine growing season) from 2015 to 2023 (**B**) and maximum wind speed recorded from 2015 to 2023 (**C**). All the data presented are the daily averages of measurements taken every 60 min, 24 h a day, by the meteorological station located near the vineyard.

The experiment was carried out over two consecutive seasons, 2021–2022 (2022) and 2022–2023 (2023), in an experimental vineyard in the windy location of Casa de Piedra (38°09′10″ S, 67°09′20″ W and 405 m a.s.l.), La Pampa, Argentina (Figure A1A). Figure A1 shows the average wind speed and direction data collected from 2015 during the grapevine growing season (September to March), obtained from automatic meteorological stations (iMetos II, Pessl Instruments GmbH, Weiz, Austria) situated near the experimental site. Given that southwesterly winds are both the most frequent and intense, a 13-m-high windbreak poplar tree fence has been strategically positioned perpendicular to this prevailing wind direction within the vineyard. The treatments were performed in two vineyard plots: one with a selected clone of *Vitis vinifera* L. cv. Malbec (Mb), and the other with *Vitis vinifera* L. cv. Cabernet Sauvignon (CS).

**Figure A2:**
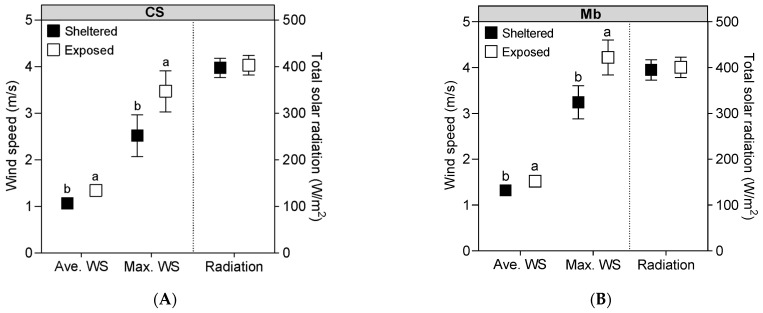
Wind speed (WS) and total solar radiation in sheltered and exposed sites for Cabernet Sauvignon (CS; (**A**)) and Malbec (Mb; (**B**)). Daily averages measured (from 8 a.m. to 8 p.m.) and repeated every 10 days during the months of grapevine growing season of 2023. Values represent means for each factor ±SEM, with different letters indicating significant differences (Fisher’s LSD, *p* ≤ 0.05).

Both cultivars were planted in 2011 on their own roots, trained using a vertical trellis system, and arranged in north–south oriented rows spaced 2.20 m apart, with 1 m between plants within each row. In each plot, vines situated from 15 m to 21 m away from the poplar windbreak were designated as sheltered, while vines positioned from 37 m to 43 m east of the sheltered site were classified as exposed. Within each wind exposure category, half of the vines were maintained without water deficit throughout the entire growing season using a drip irrigation system equipped with two drip lines (WW; well-watered treatment). Conversely, the remaining half received deficit irrigation (DI) with water supplied through only one drip line (i.e., 50% of WW). During the 2022 season, vines were well watered from budburst to veraison, and DI was applied from veraison to harvest (DIV treatment), while during the 2023 season, DI was applied from budburst to harvest (DIB treatment). In summary, with two levels of wind exposure (WE; sheltered and exposed) and two levels of irrigation (well-watered and moderate deficit irrigation), four combined treatments were implemented in each season for each cultivar. Figure A2 shows wind speed and total solar radiation measured for one day (from 8 a.m. to 8 p.m.) and repeated every 10 days, using sensors (weather station WH2900, Shenzhen Fine Offset Electronics Co., Shenzhen, China) positioned in the middle of the sheltered and exposed sites at a height of 2 m above ground level (20 cm above the canopy). While significant differences in wind speed were observed between sheltered and exposed CS and Mb vines, there were no effects on total solar radiation. A multifactorial experimental design was used, with treatments arranged in a split-plot design with 6 replicates (n = 6). The main plot encompassed wind exposure levels, while sub-plots represented irrigation levels (with 7 buffer vines maintained between irrigation treatments within a row). Each experimental unit consisted of two selected plants (based on homogeneity) from 7 consecutive plants in a row.

### 4.2. Vegetative Growth and Physiological Parameters

Shoot length and leaf area were measured at harvest from two shoots per experimental unit, according to Berli et al. [38]. Stomatal conductance (gs) was assessed at harvest in mature leaves (13th leaf from the shoot apex), fully exposed to sunlight, utilizing a steady-state diffusion leaf porometer (conductometer SC-1, Decagon Devices, Inc., Pullman, WA, USA).

### 4.3. Berry Sampling, Total Soluble Solids, Skin Phenolic Contents, Yield Component and Biomass Accumulation

At veraison and at harvest (24 °Brix), 20 berries per experimental unit were collected into nylon bags (5 berries per bunch; two from the top, two from the middle and one from the bottom). These samples were immediately placed on dry ice to prevent enzyme degradation and dehydration, then transported to the laboratory where berry fresh weight (FW) was determined before storage at −20 °C. Upon defrosting at room temperature, the berries were manually peeled. Total soluble solids (TSS) were determined using a Pocket PAL-1 digital handheld refractometer (Atago Co., Ltd., Tokyo, Japan), according to Berli et al. [39].

Berry skins were immersed in an aqueous ethanolic solution (12% ethanol, 6 g L^−1^ tartaric acid and pH 3.2) and heated at 70 °C for 3 h in darkness to determine the concentration of total anthocyanins and total phenolics index (TPI), according to Berli et al. [39].

At harvest, two bunches per experimental unit were collected in nylon bags and weighed for FW. Afterward, the berries within each bunch were detached from the rachis and sieved using stainless-steel mesh sieves with different hole diameters (10 mm, 12 mm, and 14 mm). Subsequently, the berries were counted in each category (<10 mm, 10–12 mm, 12–14 mm, and >14 mm).

The partitioning of biomass in the shoots to different organs was evaluated at harvest. One shoot per experimental unit was cut from the base and placed in a sealed plastic bag. The different shoot organs (leaves, laterals, bunches and canes) were then separated and dried in an oven at 60 °C until a constant dry weight (DW) was achieved.

### 4.4. Statistical Analysis

Statistical analysis was performed by multifactorial ANOVA with split-plot design, and pairwise comparisons were performed using LSD of Fisher test (InfoStat version 2020, Grupo InfoStat, FCA, Universidad Nacional de Córdoba, Argentina). Significance was determined at *p* ≤ 0.05. Linear regression models were calculated to evaluate the relationships between total anthocyanins and TSS, and GraphPad Prism (version 9.3.1, 2021) was used to compare the slopes of the regression lines with t-tests. Additionally, wind speed and wind direction were visualized using the Openair R package in RStudio software (version 2022.07.2).

## 5. Conclusions

Our study has confirmed our initial hypotheses, demonstrating that grapevines exposed to moderate to strong winds and deficit irrigation show reduced vegetative growth and gas exchange rates. We also found that combined stressful conditions increased the proportion of smaller berries within the bunches and enhanced biomass partition across the shoot to fruits in Malbec. Additionally, an additive increment in the content of berry skin total phenolics was observed, with these effects being more pronounced in the Malbec cultivar. These findings emphasize the importance of considering the interaction between environmental and cultural factors in grapevine production and suggest the need for management strategies for each variety based on vineyard conditions.

## Data Availability

Data is contained within the article.
